# Determining the Relationship Between Hyoid Bone Kinematics and Airway Protection in Swallowing

**DOI:** 10.1044/2021_JSLHR-21-00238

**Published:** 2022-01-04

**Authors:** Sana Smaoui, Melanie Peladeau-Pigeon, Catriona M. Steele

**Affiliations:** a Swallowing Rehabilitation Research Laboratory, KITE Research Institute – Toronto Rehabilitation Institute – University Health Network, Ontario, Canada; b Rehabilitation Sciences Institute, Temerty Faculty of Medicine, University of Toronto, Ontario, Canada

## Abstract

**Purpose:**

Research remains equivocal regarding the links between hyoid movement and penetration–aspiration. The aims of this study were (a) to explore associations between hyoid parameters, laryngeal vestibule closure (LVC) parameters, and penetration–aspiration on thin liquids; and (b) to determine which of these parameters are the strongest predictors of penetration–aspiration.

**Method:**

This study involved retrospective analysis of an existing videofluoroscopy data set, collected in 305 participants (152 males) with noncongenital/nonsurgical/non-oncological risk for dysphagia. We extracted data for six thin liquid swallows per participant, and obtained measures of hyoid movement (peak position, speed) and LVC (complete/incomplete, timing, duration). Resulting values were coded as typical/atypical relative to healthy reference data. Relationships were explored using chi-square tests and odds ratios (a) for the entire data set and (b) for the subset of data with complete LVC. Hierarchical logistic regression models determined the strongest predictors of penetration–aspiration.

**Results:**

Significant associations were found between penetration–aspiration and incomplete LVC, prolonged time-to-most-complete-LVC, short LVC duration, reduced anterior hyoid peak position, and reduced hyoid speed. Hyoid measures were also significantly associated with LVC parameters. In the first regression model, incomplete LVC and prolonged time-to-most-complete-LVC were the only significant predictors of penetration–aspiration. For cases with complete LVC, the only significant predictor was prolonged time-to-most-complete-LVC.

**Conclusions:**

Although reduced anterior hyoid peak position and speed are associated with penetration–aspiration on thin liquids, these measures do not independently account for penetration–aspiration when considered in conjunction with measures of LVC. When identifying mechanisms explaining penetration–aspiration, clinicians should focus on LVC (complete/incomplete) and timeliness of LVC.

Oropharyngeal dysphagia is characterized by impairments in airway protection and/or bolus clearance. When airway protection is inadequate, material such as food or liquid may be penetrated into the laryngeal vestibule or aspirated below the true vocal folds into the trachea. Evidence of aspiration during instrumental assessments of swallowing has been associated with increased odds of developing respiratory sequelae including pneumonia (Daniels et al., 2000; Holas et al., 1994; Ickenstein et al., 2010; Langmore et al., 1998; Macht et al., 2011; Martino et al., 2005; Pikus et al., 2003; Schmidt et al., 1994; Suntrup-Krueger et al., 2018; Walter et al., 2007). In healthy swallowing, airway protection is achieved through a number of biomechanical events leading to laryngeal vestibule closure (LVC). These include arytenoid adduction, anterior movement of the arytenoid cartilages to achieve contact with the epiglottic petiole, laryngeal elevation, and epiglottic inversion (Ekberg, 1982; Inamoto et al., 2011; Kawasaki et al., 2001; Logemann et al., 1992; Ohmae et al., 1995; Sasaki & Weaver, 1997; Shaker et al., 1990; Vose & Humbert, 2019). In situations where LVC is compromised (i.e., incomplete), material may enter the vestibule and travel as far as the vocal folds (i.e., penetration), or below the vocal folds into the trachea (i.e., aspiration; Kahrilas et al., 1997; Rosenbek et al., 1996). Furthermore, even when LVC is not compromised, delays in the timing of LVC (i.e., prolonged time-to-LVC) may afford an opportunity for airway invasion (Barbon et al., 2020; Vose & Humbert, 2019; Waito et al., 2020). A third parameter of potential relevance is the duration of LVC, once achieved. Early termination of LVC (i.e., short LVC duration) is not a common observation in poststroke dysphagia (Park et al., 2010), but has recently been described as a characteristic of swallowing in individuals with chronic obstructive pulmonary disease (Mancopes et al., 2020). Short LVC duration may increase the risk of airway invasion after the swallow, particularly if there is residue in the pharynx when the airway opens (Steele et al., 2020). Interestingly, recent studies have shown that LVC duration increases with healthy aging (Mancopes et al., 2021), suggestive of a possible compensation to maintain airway protection in the context of slower bolus clearance.

It has been suggested that movement of the hyoid bone plays a role in the biomechanical events leading to LVC. Hyoid movement occurs in response to suprahyoid muscle contraction. The onset of this movement (referred to as the “hyoid burst”) is characterized by a rapid jump in the anterior–superior direction, which generates traction forces on the larynx and upper esophageal sphincter, thus functioning as a pulley (Mendelsohn & McConnel, 1987; Pearson et al., 2011; Steele et al., 2011). Hyoid parameters that have been studied include both temporal and kinematic measures: time-to-peak position, time-at-peak-position, duration of hyoid return-to-rest (Lof & Robbins, 1990; Molfenter & Steele, 2014a; Nagy et al., 2014, 2015; Ragland et al., 2016; Zoratto et al., 2010) and peak position, displacement or excursion distance, and velocity or speed (Dodds et al., 1988; Kim & McCullough, 2008, 2010; Leonard et al., 2000; Logemann et al., 2000; Paik et al., 2008; Perlman et al., 1995; Steele et al., 2011). Kinematic measures are typically reported along the anterior (*x*), superior (*y*), and hypotenuse (*xy*) axes of movement, relative to a *y*-axis defined parallel to the cervical spine (Molfenter & Steele, 2011, 2014b; Perlman et al., 1995). Recent reference values for anatomically scaled measures of peak *xy* hyoid position and hyoid *xy* speed (i.e., the rate of change in position along the hypotenuse axis) have been reported for healthy adults under the age of 60 years (Smaoui et al., 2021) and show greater displacement (relative to the anterior inferior corner of C4) and faster speed with larger boluses, but no significant differences across the range from thin to extremely thick liquid consistencies. A subsequent paper, including data for an additional cohort of healthy adults, aged 60–82 years, found no significant effects of age on hyoid parameters, suggesting that the reference values reported for adults under age 60 years can be used when evaluating hyoid movement in older individuals (Mancopes et al., 2021).

The literature contains mixed information regarding the question of whether abnormalities in hyoid movement are associated with penetration–aspiration. A review by Steele and Cichero (2014) concluded that penetration–aspiration was associated with reduced hyoid excursion and impairments in parameters characterizing LVC. In contrast to these findings, Molfenter and Steele (2014a) were unable to find differences in hyoid excursion between aspirators and nonaspirators in a study of patients with neurogenic dysphagia of heterogeneous etiology. In another study by Steele et al. (2011), individuals with the greatest reductions in anterior hyoid excursion (i.e., below the first quartile boundary for their sample) had significantly higher rates of penetration–aspiration. Finally, in two papers arising from a recent study exploring relationships between hyoid displacement and risk of penetration–aspiration, Zhang et al. (2020, 2021) found that reduced anterior hyoid bone displacement (but not other hyoid movement parameters) predicted penetration–aspiration in a cohort of patients with mixed dysphagia.

Although impaired hyoid movement is often seen in patients with dysphagia (e.g., Kendall & Leonard, 2001; Kim & McCullough, 2010; Lee et al., 2021; Paik et al., 2008; Wang et al., 2010), precise measurement of hyoid bone kinematics during videofluoroscopy analysis is time consuming. Unfortunately, recent research suggests that the alternative, namely, visuoperceptual judgment of hyoid movement, lacks sensitivity compared to quantitative measurements (Brates et al., 2019, 2020). It is, therefore, important to confirm whether measures of hyoid movement are value added and provide important information to identify and elucidate the mechanisms behind impaired swallowing safety (i.e., penetration–aspiration). It is also important to determine whether hyoid measures provide information that is different from, or overlapping with, measures of LVC (complete/incomplete), timing, and duration, so that decisions can be made about the value of measuring one versus multiple parameters.

Therefore, the overarching goal of this article was to determine whether hyoid parameters should be routinely examined when evaluating swallowing safety. To achieve this aim, we sought to answer these specific questions:

1. Are there significant associations between penetration–aspiration and
   a. atypical hyoid peak position (*x*, *y*, and *xy* coordinates) or speed;
   b. atypical measures of LVC (i.e., incomplete), LVC timing, and/or LVC duration?
2. Are there significant associations between atypical hyoid peak position (*x*, *y*, and *xy* coordinates) or speed and incomplete, delayed, or short LVC?
3. When considering hyoid peak position (*x*, *y*, and *xy* coordinates), hyoid speed, LVC (complete/incomplete), LVC timing, and LVC duration, which parameter(s) is/are the best predictor(s) of penetration–aspiration?

For the remainder of this article, the term *hyoid peak position* will be used to refer to the furthest position of the hyoid bone along the *xy* hypotenuse axis from the anterior inferior corner of C4, expressed in *x* (anterior), *y* (superior), or *xy* (hypotenuse) coordinates. The term *hyoid speed* will be used to refer to the rate of change in hyoid position along the *xy* hypotenuse axis between onset of the hyoid burst and the first frame at peak position. Figure 1 provides an example of two videofluoroscopic images from the same person, captured on the frames of hyoid burst and hyoid peak position, with the *x*, *y*, and *xy* axes of measurement and the cervical spine scalar labelled.

**Figure 1.**
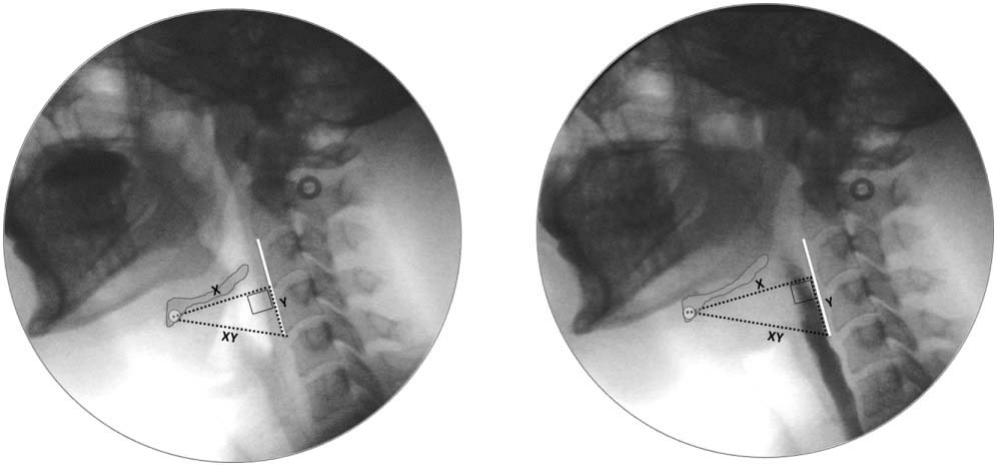
Videofluoroscopic images taken from the same person on the frames of hyoid burst (left) and peak hyoid position (right). The images show the *x* (anterior), *y* (superior), or *xy* (hypotenuse) axes of measurement, used to determine the position of the hyoid relative to the anterior–inferior corner of C4. The white lines illustrate the C2–C4 cervical spine scalar used to convert position measurements in pixels to anatomically scaled units, that is, %(C2–C4). Hyoid speed is calculated based on the difference in *xy* position between the frames of hyoid burst and peak hyoid position, divided by the time difference between these frames.

## Method

### Participants

This study involved secondary analysis of an existing data set and was approved by the University Health Network Research Ethics Board (CAPCR #13–6890). The original data were collected in an industry-funded study (Steele, Mukherjee, et al., 2019) of 305 participants (152 males) who were considered at-risk for noncongenital, nonsurgical, and non-oncological oropharyngeal dysphagia. This included individuals with primary diagnoses of stroke or acquired brain injury, and other adults aged 50 years or older with signs or symptoms of dysphagia. Participants were recruited from seven hospitals between November 7, 2013, and March 11, 2015. Exclusion criteria included (a) prior surgery to the neck, (b) presence of a nasogastric feeding tube, (c) nonsurgical trauma to the neck leading to musculoskeletal or nerve injury, and (d) radiation to the neck. Additional details can be found in the original article (Steele, Mukherjee, et al., 2019).

### Videofluoroscopy and Rating Procedures

The original study involved a videofluoroscopy (videofluoroscopic swallowing study [VFSS]), which began with up to six naturally sized cup sips of Bracco Varibar Thin Liquid barium diluted to a 20% weight-to-volume concentration. VFSS recordings were collected in lateral view at 30 frames per second, stripped of audio, and spliced into clips, each containing the swallows for a single bolus. In total, 1,730 thin liquid bolus clips were available; these were assigned randomly for blinded rating, in duplicate, by a team of trained raters using the ASPEKT method (Analysis of Swallowing Physiology: Events, Kinematics and Timing; Steele, Peladeau-Pigeon, et al., 2019), a stepwise procedure that involves

1. counting the number of swallows per bolus;
2. scoring penetration–aspiration on each swallow using the 8-point Penetration–Aspiration Scale (PAS; Rosenbek et al., 1996);
3. identifying the frame numbers of key events in the swallowing sequence, from which timing measures are derived; and
4. measuring structural movement, anatomical area, or residue area on key frames using anatomically scaled pixel-based measures in Image-J open-source software (National Institutes of Health).

Interrater agreement was calculated across independent duplicate ratings, and disagreements between raters were taken to a consensus meeting for review and resolution. Thresholds for discrepancy and rules for frame selection were based on methods outlined in the supplemental material published by Steele, Peladeau-Pigeon, et al. (2019). This rating procedure yielded the following measures for each bolus:

(a)  Ratings of maximum PAS score seen across the swallows for each bolus, and transformed into a binary classification of “safe” versus “unsafe,” that is, < versus ≥ 3.
(b)  Ratings of LVC as complete or incomplete.
(c)  Measures of time-to-most-complete-LVC, which has previously been termed *laryngeal vestibule reaction time* (i.e., the interval between onset of the hyoid burst and the first frame of most-complete LVC; note, in the case of incomplete LVC, the interval between onset of the hyoid burst and the frame of laryngeal vestibule approximation [i.e., the closest approximation of the arytenoid cartilages to the epiglottis] was calculated).
(d)  Measures of LVC duration (i.e., the interval between the first frame of LVC and the first frame of laryngeal vestibule opening; note, in the case of incomplete LVC, a missing data point was entered for LVC duration).

The protocol for the previous study also included boluses of thickened liquids; however, penetration–aspiration was most common on the thin liquid stimuli (Steele, Mukhurjee, et al., 2019); therefore, for this retrospective analysis, only the thin liquid trials were used.

Hyoid position was tracked frame-by-frame, starting 10 frames before the onset of the hyoid burst and continuing until 10 frames after the hyoid returned to rest after completion of the swallow. The *x*, *y*, and *xy* coordinates of hyoid position were recorded in anatomically normalized units, that is, %(C2–C4), relative to the anterior–inferior corner of C4, with the *y*-axis of the coordinate system defined by the C2–C4 cervical spine (Molfenter & Steele, 2014b). Illustrations of this method can be found in prior publications (Molfenter & Steele, 2014b; Steele, Peladeau-Pigeon, et al., 2019). Missing data points were recorded by raters if the hyoid bone moved out of the field of view or if visualization of the cervical spine was obstructed by the participant's shoulder. The frame of hyoid peak position was identified from the time history of these measurements. Hyoid speed was calculated as the ratio of distance travelled from the position on the frame of hyoid burst onset to the first frame of hyoid peak position, divided by movement duration. Duplicate hyoid tracking was performed on a randomly selected subset of 40% of the thin boluses for the purposes of determining reliability.

### Analyses

Statistical analyses were conducted using IBM SPSS statistics Version 27. Interrater reliability for binary PAS scores and ratings of LVC were evaluated based on the proportion of preconsensus ratings displaying absolute agreement. Interrater agreement for the identification of frames for key events, from which timing measures were derived, was explored by determining the proportion of preconsensus ratings falling with a difference of ≤ 3 frames. For measures of hyoid peak position and speed, interrater agreement was calculated using average intraclass correlations (ICCs) for consistency based on a two-way mixed-effects model.

For the analyses described in this article, boluses were only included if all measures were available. Descriptive statistics were computed for all variables. The relationships between predictor parameters were then explored using Pearson correlations. Correlations of *R* > .3 were identified between the two continuous LVC parameters (time-to-most-complete-LVC and LVC duration), and between the four hyoid variables (the *x*, *y*, and *xy* coordinates of hyoid peak position, and hyoid speed). These findings prompted exploratory multivariate linear regression models incorporating correlated predictors and an outcome variable of PAS < versus ≥ 3, to test for multicollinearity, based on a variance inflation factor > 10. These explorations confirmed multicollinearity within the hyoid peak position parameters, which was resolved by limiting these predictors to the *x* and *y* (but not the *xy*) coordinates of peak position. Based on these preliminary explorations of the data, we decided to apply Bonferroni corrections to the alpha criteria for the subsequent analyses, as follows: *p* < .025 for the LVC parameters and *p* < .017 for the hyoid parameters.

The raw hyoid and LVC parameter data were then classified in a binary fashion as either typical (i.e., within the healthy interquartile range) or atypical (< the 25th percentile or > the 75th percentile) based on published reference values for healthy participants (Smaoui et al., 2021; Steele, Peladeau-Pigeon, et al., 2019; Steele Swallowing Lab, 2021). We were able to obtain previously unpublished reference values for the 25th percentile *x* and *y* coordinates of peak hyoid position from the data set used previously by (Smaoui et al., 2021). Table 1 summarizes the reference values used for classifying each parameter as atypical.

**Table 1.** Reference values used for classifying each parameter as atypical.

Variable	Definition of atypical values
PAS score (unsafe)	3 or higher
LVC integrity	Incomplete
Time-to-most-complete-LVC	> 259 ms
LVC Duration	< 367 ms
Hyoid peak position (*x* coordinate)	< 133%(C2–C4)
Hyoid peak position (*y* coordinate)	< 74%(C2–C4)
Hyoid speed (burst onset to peak)	< 96%(C2–C4)/s

*Note.* Source: Steele, Peladeau-Pigeon, et al. (2019). LVC = laryngeal vestibule closure; ms = milliseconds.

Cross-tabulations, chi-square tests, and odds ratios were performed to test the associations between atypical values for hyoid and LVC parameters, and penetration–aspiration. Given that measures of LVC duration are only valid in the context of complete LVC, these tests of association were explored first for the entire data set (excluding LVC duration), and then for the subset of data where LVC was complete (including LVC duration). For the chi-square tests, a *p* value of < .05 was considered statistically significant. For the odds ratios, values with lower 95% confidence interval boundaries > 1.0 were considered significant.

Two hierarchical logistic regression models were then built to determine which of the individual or combined hyoid and/or LVC parameters were significant predictors of penetration–aspiration. The first model explored these parameters in the entire data set, excluding the LVC duration predictor. The second model explored these parameters in the subset of data with complete LVC, and included LVC duration as a predictor. Assumptions of linearity were confirmed using the Box-Tidwell approach.

## Results

### Interrater Reliability

The proportion of ratings showing agreement, prior to the resolution of discrepancies by consensus was 97% for binary PAS scores, and 91% for judgments of LVC (complete vs incomplete). Interrater agreement within 3 frames was seen on 93% of ratings identifying the frame of hyoid burst onset, 63% of ratings identifying the frame of LVC, and 93% of ratings identifying the frame of LVC offset. For measures of hyoid peak *xy* position, derived from the frame-by-frame time histories of hyoid position tracking, an ICC of 0.939 (95% confidence interval 0.929 to 0.948) was found, indicating excellent reliability.

### Descriptive Statistics

Table 2 displays the descriptive statistics for the continuous parameters in this data set. After removal of boluses where data were missing (due to issues with image quality, shadows, or positioning that interfered with the measurement of one or more parameters), the data set comprised 1,224 thin liquid swallows. LVC was judged to be complete on 1,176 of these swallows, and incomplete on 48 (4%). Results will be presented first for the entire data set, and second for the LVC-complete subset.

**Table 2.** Descriptive statistics for the continuous parameters.

Parameter	Unit	*M*	95% Confidence interval of the mean		*SD*
			Lower bound	Upper bound	
Time-to-most-complete-LVC	ms	116	102	131	256
LVC duration^a^	ms	623	607	638	280
Hyoid peak position (*x* coordinate)	%(C2–C4)	155%	154%	157%	26%
Hyoid peak position (*y* coordinate)	%(C2–C4)	102%	100%	103%	30%
Hyoid speed	%(C2–C4)/s	116%	113%	120%	61%

*Note.* LVC = laryngeal vestibule closure; ms = milliseconds.

a LVC duration was calculated only on the subset of data with complete LVC.

### Results for the Entire Data Set

Table 3 shows the frequencies of typical versus atypical values for the parameters of interest, for the overall data set.

**Table 3.** Bolus-level frequencies of typical versus atypical values for each parameter of interest for the entire data set (*N* = 1,224 swallows).

Parameter	Typical values	Atypical values
	*n* (%)	*n* (%)
PAS	1,157 (95)	67 (5)
LVC integrity	1,176 (96)	48 (4)
Time-to-most-complete-LVC	945 (77)	279 (23)
LVC duration	1,075 (91)	101 (9)
Hyoid peak position (*x* coordinate)	1,045 (85)	179 (15)
Hyoid peak position (*y* coordinate)	1,022 (84)	202 (17)
Hyoid speed	717 (59)	507 (41)

*Note.* PAS = Penetration–Aspiration Scale; LVC = laryngeal vestibule closure.

#### Question 1: Associations Between Hyoid Parameters, LVC Parameters, and Penetration–Aspiration

Table 4 shows the results of the chi-square and odds ratio statistics exploring associations between hyoid parameters, LVC parameters and penetration–aspiration for the entire data set. The order of listing in the table reflects the strength of the observed associations: the strongest associations with penetration–aspiration were seen with incomplete LVC and prolonged time-to-most-complete-LVC. Reduced anterior hyoid peak hyoid position, below the 25th percentile reference value, and reduced hyoid speed, were both significantly associated with penetration–aspiration, while reduced superior hyoid peak position was not.

**Table 4.** Chi-square and odds ratios for the associations between hyoid parameters, LVC parameters, and swallowing safety for the entire data set.

Dependent variable	Independent variable	Chi-square	*p*	Odds ratio	95% Confidence interval	
					Lower bound	Upper bound
PAS (safe vs. unsafe)	Partial or incomplete LVC	157.28	< .001	21.27	11.20	40.38
	Prolonged time-to-most-complete-LVC	59.39	< .001	6.09	3.65	10.16
	Reduced hyoid peak position: *x* coordinate	6.56	.01	2.09	1.18	3.71
	Reduced hyoid peak position: *y* coordinate	0.1	.75	1.11	0.58	2.11
	Reduced hyoid speed	5.57	.02	1.80	1.10	2.96
Incomplete LVC	Reduced hyoid peak position: *x* coordinate	17.3	< .001	3.43	1.86	6.35
	Reduced hyoid peak position: *y* coordinate	4.06	.04	1.94	1.01	3.74
	Reduced hyoid speed	11.05	< .001	2.68	1.47	4.90
Prolonged time-to-most-complete-LVC	Reduced hyoid peak position: *x* coordinate	21.77	< .001	2.21	1.58	3.11
	Reduced hyoid peak position: *y* coordinate	16.24	< .001	1.95	1.40	2.71
	Reduced hyoid speed	26.81	< .001	2.03	1.55	2.66

*Note.* PAS = Penetration–Aspiration Scale; LVC = laryngeal vestibule closure.

#### Question 2: Associations Between Hyoid Parameters and LVC Parameters

With respect to associations between the hyoid parameters and the LVC parameters for the entire data set, reductions in all three hyoid parameters were significantly associated both with incomplete LVC and with prolonged time-to-most-complete-LVC

#### Question 3: Predictors of Penetration–Aspiration

Predictors were added to the hierarchical logistic regression models in descending order of the strength of the chi-square results reported above (see Table 4). For Regression Model 1, including all data, the order was: (a) LVC (complete/incomplete); (b) time-to-most-complete-LVC; (c) hyoid *xy* speed; (d) hyoid peak position (*x* coordinate); and (e) hyoid peak position (*y* coordinate). The results can be found in Table 5. Only LVC and time-to-most-complete-LVC were found to be significant predictors of unsafe swallowing. The presence of incomplete LVC and prolonged time-to-most-complete-LVC explained 21% of the variance in penetration–aspiration, and contributed to 11.68- and 3.85-fold odds of penetration–aspiration, respectively. The addition of hyoid parameters to Model 1 did not significantly improve prediction.

**Table 5.** Hierarchical logistic regression model results for predictors of penetration–aspiration (data subset with complete LVC).

Step	Omnibus Test (chi-square)	Significance	Variance explained (Nagelkerke *R* ^2^)	Predictor	Wald chi-square by component	Significance	Exponent	95% Confidence interval	
								Lower bound	Upper bound
1	71.41	< .001	.16	LVC integrity = incomplete	87.33	< .001	21.27	11.2	40.38
2	21.43	< .001	.21	LVC integrity = incomplete	48.98	< .001	11.68	5.87	23.24
				Time-to-most-complete-LVC (prolonged)	22.32	< .001	3.85	2.2	6.73
3	93.19	< .001	.21	LVC integrity = incomplete	47.47	< .001	11.4	5.71	22.79
				Time-to-most-complete-LVC (prolonged)	20.98	< .001	3.75	2.13	6.6
				Hyoid speed (reduced)	0.36	0.55	1.18	0.68	2.04
4	93.34	< .001	.21	LVC integrity = incomplete	46.11	< .001	11.2	5.58	22.5
				Time-to-most-complete-LVC (prolonged)	20.48	< .001	3.71	2.1	6.55
				Hyoid speed (reduced)	0.25	0.62	1.15	0.66	2.03
				Hyoid peak position: *x* coordinate (reduced)	0.16	0.69	1.15	0.58	2.26
5	94.72	< .001	.22	LVC integrity = incomplete	46.80	< .001	11.49	5.71	23.14
				Time-to-most-complete-LVC (prolonged)	21.65	< .001	3.87	2.19	6.85
				Hyoid speed (reduced)	0.26	0.54	1.16	0.66	2.03
				Hyoid peak position: *x* coordinate (reduced)	0.37	0.26	1.24	0.62	2.45
				Hyoid peak position: *y* coordinate (reduced)	1.29	0.97	0.65	0.31	1.37

*Note.* LVC = laryngeal vestibule closure.

### Results for the Subset of Data With Complete LVC

Table 6 shows the frequencies of typical versus atypical values for the parameters of interest, for the swallows with complete LVC.

**Table 6.** Bolus-level frequencies of typical versus atypical values for each parameter of interest for the subset of data with complete LVC (*N* = 1,176 swallows).

Parameter	Typical values	Atypical values
	*n* (%)	*n* (%)
LVC duration	1,075 (91)	101 (9)
Hyoid peak position (*x* coordinate)	1,014 (86)	162 (14)
Hyoid peak position (*y* coordinate)	987 (84)	189 (16)
Hyoid speed	700 (60)	476 (40)

*Note.* LVC = laryngeal vestibule closure.

#### Question 1: Associations Between Hyoid Parameters, LVC Parameters, and Penetration–Aspiration

Table 7 shows the results of the chi-square and odds ratio statistics exploring associations between hyoid parameters, LVC parameters, and penetration–aspiration for the subset of the data with complete LVC. The order of listing in the table reflects the strength of the observed associations: The strongest associations with penetration–aspiration were seen with prolonged time-to-most-complete-LVC and short LVC duration. Reductions in hyoid peak position (either anterior or superior), or in hyoid speed were not significantly associated with penetration–aspiration in this subset of the data.

**Table 7.** Chi-square and odds ratios for the associations between hyoid parameters, LVC parameters, and swallowing safety for the subset of the data with complete LVC.

Dependent variable	Independent variable	Chi-square	*p* value	Odds ratio	95% Confidence interval	
					Lower bound	Upper bound
PAS (safe vs. unsafe)	Prolonged time-to-most-complete-LVC	34.75	< .001	5.24	2.86	9.60
	Short LVC duration	5.03	.03	2.41	1.09	5.33
	Reduced hyoid peak position: *x* coordinate	1.53	.22	1.60	0.76	3.38
	Reduced hyoid peak position: *y* coordinate	0.1	.75	1.14	0.52	2.48
	Reduced hyoid speed	3.2	.07	1.72	0.94	3.12
Prolonged time-to-most-complete-LVC	Reduced hyoid peak position: *x* coordinate	13.41	< .001	1.97	1.36	2.84
	Reduced hyoid peak position: *y* coordinate	11.04	< .001	1.80	1.27	2.56
	Reduced hyoid speed	18.92	< .001	1.87	1.41	2.49
Short LVC duration	Reduced hyoid peak position: *x* coordinate	2.36	.13	1.51	0.89	2.56
	Reduced hyoid peak position: *y* coordinate	7.66	.006	1.94	1.21	3.12
	Reduced hyoid speed	0.2	.65	1.10	0.73	1.66

*Note.* PAS = Penetration–Aspiration Scale; LVC = laryngeal vestibule closure.

#### Question 2: Associations Between Hyoid Parameters and LVC Parameters

With respect to associations between the hyoid parameters and the LVC parameters in the subset of data with complete LVC, reductions in all three hyoid parameters were significantly associated with prolonged time-to-most-complete-LVC. A significant association was also found between reduced superior hyoid peak position and short LVC duration.

#### Question 3: Predictors of Penetration–Aspiration

As with the previous model, predictors were added to the second hierarchical logistic regression model (for the subset of data with complete LVC) in descending order of the strength of the chi-square results reported in Table 7: (a) time-to-most-complete-LVC; (b) LVC duration; (c) hyoid *xy* speed; (d) hyoid peak position (*x* coordinate); and (e) hyoid peak position (*y* coordinate). The results can be found in Table 8. Prolonged time-to-most-complete-LVC was the only statistically significant predictor of penetration–aspiration. Values above the 75th percentile boundary of the healthy reference distribution, accounted for 8% of the observed variance and led to a 5.24-fold increase in the odds of penetration–aspiration. The addition of LVC duration or hyoid parameters to the model did not significantly improve model prediction.

**Table 8.** Hierarchical logistic regression model results for predictors of penetration–aspiration (entire data set).

Step	Omnibus Test (chi-square)	Significance	Variance explained (Nagelkerke *R* ^2^)	Predictor	Wald chi-square by component	Significance	Exponent	95% Confidence interval	
								Lower bound	Upper bound
1	27.63	< .001	.08	Time-to-most-complete-LVC (prolonged)	28.64	< .001	5.24	2.86	9.6
2	27.74	< .001	.08	Time-to-most-complete-LVC (prolonged)	25.77	< .001	5.47	2.84	10.54
				LVC duration (reduced)	0.11	0.74	0.87	0.36	2.05
3	28.81	< .001	.09	Time-to-most-complete-LVC (prolonged)	23.39	< .001	5.16	2.65	10.03
				LVC duration (reduced)	0.06	0.80	0.89	0.38	2.13
				Hyoid speed (reduced)	1.07	0.30	1.38	0.75	2.55
4	28.88	< .001	.09	Time-to-most-complete-LVC (prolonged)	23.03	< .001	5.12	2.63	9.98
				LVC duration (reduced)	0.07	0.80	0.89	0.38	2.13
				Hyoid speed (reduced)	0.84	0.59	1.35	0.71	2.56
				Hyoid peak position: *x* coordinate (reduced)	0.08	0.78	1.12	0.50	2.50
5	28.99	< .001	.09	Time-to-most-complete-LVC (prolonged)	23.20	< .001	5.16	2.65	10.06
				LVC duration (reduced)	0.05	0.82	0.91	0.38	2.16
				Hyoid speed (reduced)	0.86	0.35	1.35	0.71	2.57
				Hyoid peak position: *x* coordinate (reduced)	0.10	0.75	1.14	0.51	2.55
				Hyoid peak position: Y coordinate (reduced)	0.11	0.75	0.87	0.39	1.97

*Note.* LVC = laryngeal vestibule closure.

## Discussion

The analyses in this article elucidate the utility of hyoid measurement and its relationship to LVC parameters and swallowing safety as measured by the PAS. We had expected to see associations between atypical values for hyoid parameters, atypical values for LVC parameters, and penetration–aspiration in this cohort. Our findings suggest that there are indeed associations between hyoid parameters and LVC, and between hyoid parameters and penetration–aspiration, when these relationships are explored in isolation. However, the importance of the associations between hyoid parameters and penetration–aspiration diminished in the hierarchical regression models, which found that incomplete LVC and prolonged time-to-most-complete-LVC were the best predictors of penetration–aspiration. A novel aspect of this study is the recognition that measures of LVC duration are only valid and therefore only appropriate to explore as potential predictors of penetration–aspiration, in cases with complete LVC.

Our results regarding the univariate relationships between anterior peak position and penetration–aspiration concur with those reported by Zhang et al. (2020, 2021), despite differences in analysis approach. Zhang et al. (2020, 2021) used generalized estimating equations to explore the relationship between measures of hyoid kinematics and penetration–aspiration, but did not consider other physiological parameters such as LVC. This difference in approach likely explains the differences in study results. Additionally, Zhang et al. (2020, 2021) included data for several different bolus consistencies, while our analysis focused only on thin liquids. It is important to highlight that both analyses incorporated a heterogeneous sample of patients, and a future focus of investigation should be to determine whether different results, suggesting different mechanisms of impairment, would be found across samples of patients with dysphagia of different etiologies.

### Clinical Applicability

Our findings point to the primary importance of evaluating LVC and LVC timing (as opposed to hyoid parameters) as indices of the risk of penetration–aspiration in clinical swallowing evaluations. Our findings also raise questions about the utility of automated measurement of hyoid bone kinematics (e.g., Zhang et al., 2020, 2021) as a method that may be able to detect penetration–aspiration with high sensitivity and specificity. When considering the physiological events that lead to LVC, movements of the tongue, larynx, and hyoid are all known to contribute; thus, relationships between hyoid movement and LVC parameters are to be expected. However, information about hyoid excursion alone does not appear to add meaningfully to identifying the primary physiological mechanisms that lead to penetration–aspiration. Comparison of patient LVC parameters to reference values in healthy adults enables clinicians to determine whether either the completeness of closure or timing of LVC are atypical, and therefore appropriate to target in intervention, and monitor as key outcome measures that are related to swallowing safety.

### Limitations

This study is not without limitations. One limitation was the distribution of the raw continuous data, where a skew toward typical values was seen for most parameters. These skews meant that the assumptions for parametric statistics or principal component analysis were violated and led to our decision to pursue frequency-based analysis and logistic regression using binary transformations of the raw data into typical versus atypical values.

Another challenging characteristic of the data set used in this study was the fact that the patients who presented with penetration–aspiration did not do so consistently on every single thin liquid bolus. The data set used for this study was collected from a cohort of adults who were judged to be at risk for dysphagia based on symptoms or medical history that were considered sufficient to warrant an instrumental swallowing assessment. As such, even though the data contain examples of swallows that do not involve penetration–aspiration, the study sample should not be considered to represent individuals with healthy swallowing. Variability in penetration–aspiration across boluses presents a particular challenge for statistical analysis, in that assumptions of nonindependence across boluses collected from the same individual do not appear to hold true. For this reason, as in previous studies (e.g., Molfenter & Steele, 2014a; Zhang et al., 2020, 2021), the mechanistic relationships between hyoid parameters and penetration–aspiration are analyzed at the bolus rather than the participant level in this study.

As previously noted, this analysis explored thin boluses only, given that the prevalence of penetration–aspiration in the original study was higher on thin liquids than thicker textures (Steele, Mukhurjee, et al., 2019). It remains unknown how thicker bolus consistencies would impact the model predictions seen in this analysis; however, given that thicker consistencies move more slowly through the oropharynx, it seems plausible that predictors involving timing might be impacted. In particular, time-to-most-complete-LVC (previously called LVC reaction time) has been shown to vary as a function of bolus consistency, with shorter values seen with thicker consistencies (Steele, Peladeau-Pigeon, et al., 2019).

The reference thresholds used in this analysis were based on published reference values for individuals aged below 60 years. It is acknowledged that some of these thresholds may vary slightly with healthy normal aging. However, in a recent publication (Mancopes et al., 2021), which added a cohort of healthy adults aged 60–82 years to the original cohort used for the calculation of reference values by Steele, Peladeau-Pigeon, et al. (2020), no significant age-related changes were found in time-to-LVC, hyoid *xy* peak position, and hyoid *xy* speed for thin liquid swallows. LVC duration was found to increase with healthy aging, suggesting that the threshold used to define atypical, short LVC duration would need to shift. Nevertheless, given that LVC duration was not found to be a significant predictor of penetration–aspiration for cases with complete LVC in this study, this is likely to have minimal impact.

Finally, it is possible that other parameters, which were not considered in the models in this study, may explain significant portions of the observed variance in penetration–aspiration, either independently or through interactions with LVC and hyoid measures. One parameter of potential relevance is the presence and severity of preswallow residue, which has previously been identified as a significant predictor of penetration–aspiration on subsequent swallows (Steele et al., 2020).

## Conclusions

This secondary analysis aimed to delineate the role of hyoid kinematics in unsafe swallowing. Our analyses revealed significant relationships between penetration–aspiration and reductions in both anterior hyoid excursion and hyoid *xy* speed, when examined in isolation. However, when considered in conjunction with parameters of LVC (complete/incomplete) and timing, hyoid parameters were no longer significant predictors of penetration–aspiration. Ultimately, reductions in hyoid movement are significantly associated with impairments in LVC, which are the primary predictors of unsafe PAS scores.

## Author Contributions

**Sana Smaoui:** Conceptualization (Equal), Data curation (Equal), Formal analysis (Equal), Investigation (Equal), Validation (Equal), Visualization (Lead), Writing – original draft (Lead), Writing – review & editing (Equal). **Melanie Peladeau-Pigeon:** Data curation (Equal), Investigation (Equal), Project administration (Equal), Software (Lead), Writing – review & editing (Supporting). **Catriona M. Steele:** Conceptualization (Equal), Formal analysis (Equal), Funding acquisition (Lead), Investigation (Lead), Methodology (Equal), Project administration (Equal), Supervision (Lead), Validation (Lead), Writing – original draft (Equal), Writing – review & editing (Equal).
